# Plasmon-enhanced fluorescence for biophotonics and bio-analytical applications

**DOI:** 10.3389/fchem.2024.1407561

**Published:** 2024-06-26

**Authors:** Souradip Dasgupta, Krishanu Ray

**Affiliations:** ^1^ Division of Vaccine Research, Institute of Human Virology, University of Maryland School of Medicine, Baltimore, MD, United States; ^2^ Department of Biochemistry and Molecular Biology, University of Maryland School of Medicine, Baltimore, MD, United States

**Keywords:** plasmon-enhanced fluorescence, biophotonic applications, analytical applications, radiative decay rate, fluorescence

## Abstract

Fluorescence spectroscopy serves as an ultrasensitive sophisticated tool where background noises which serve as a major impediment to the detection of the desired signals can be safely avoided for detections down to the single-molecule levels. One such way of bypassing background noise is plasmon-enhanced fluorescence (PEF), where the interactions of fluorophores at the surface of metals or plasmonic nanoparticles are probed. The underlying condition is a significant spectral overlap between the localized surface plasmon resonance (LSPR) of the nanoparticle and the absorption or emission spectra of the fluorophore. The rationale being the coupling of the excited state of the fluorophore with the localized surface plasmon leads to an augmented emission, owing to local field enhancement. It is manifested in enhanced quantum yields concurrent with a decrease in fluorescence lifetimes, owing to an increase in radiative rate constants. This improvement in detection provided by PEF allows a significant scope of expansion in the domain of weakly emitting fluorophores which otherwise would have remained unperceivable. The concept of coupling of weak emitters with plasmons can bypass the problems of photobleaching, opening up avenues of imaging with significantly higher sensitivity and improved resolution. Furthermore, amplification of the emission signal by the coupling of free electrons of the metal nanoparticles with the electrons of the fluorophore provides ample opportunities for achieving lower detection limits that are involved in biological imaging and molecular sensing. One avenue that has attracted significant attraction in the last few years is the fast, label-free detection of bio-analytes under physiological conditions using plasmonic nanoparticles for point-of-care analysis. This review focusses on the applications of plasmonic nanomaterials in the field of biosensing, imaging with a brief introduction on the different aspects of LSPR and fabrication techniques.

## 1 Introduction

**FIGURE 1:**
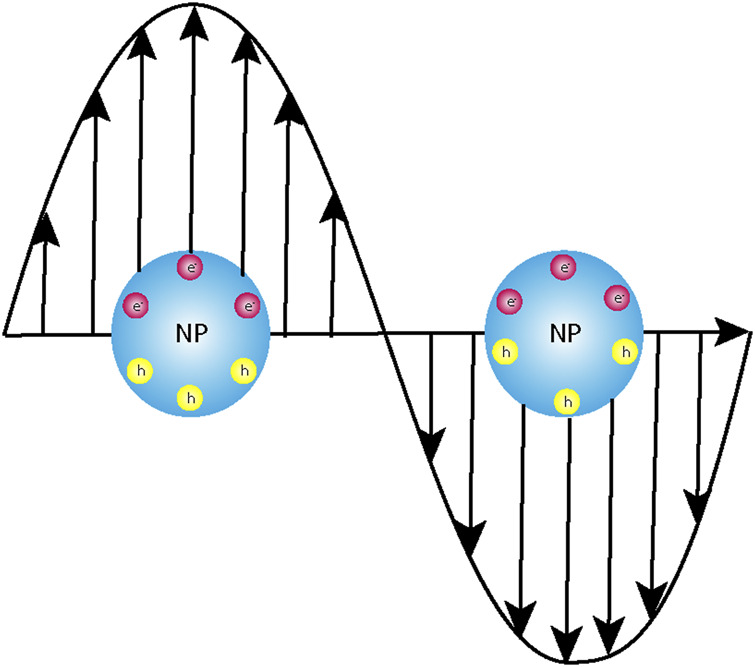
Schematic representation of the interaction of the electromagnetic light wave with the instantaneously created dipole post-photoexcitation leading to localized surface plasmon resonance (LSPR).

Light–matter interactions manifested by absorption, emission, and scattering have paved the way for the design and applications of fluorophores in a variety of real-time problems such as organic electronics, biosensors, and photocatalysis (Spitzberg et al., 2019; Rivera and Kaminer, 2020; Peng et al., 2022a; Kumar et al., 2022; Zhang et al., 2023; Zhang et al., 2024). The wavelength of light in the solar spectrum typically ranges in a few hundreds of nanometres. However, the size of the fluorogenic molecules or the plasmonic nanoparticles under investigation posed a serious lacuna to the extent of these interactions and, consequently, their applications. With the advent of nanomaterials, these could be subsided to a certain extent. When organic fluorophores are irradiated by monochromatic light, a section of the molecules lying in the ground state gets excited, and the extra energy is finally released as photons; the process is defined as photoluminescence. The augmented spectral intensity observed in metal nanoparticles is attributed to the excitation of the localized surface plasmon resonance (LSPR) that results in the higher-extinction cross sections of the plasmonic nanoparticles (Luk yanchuk et al., 2010). The above discussion necessitates a very brief overview of plasmons and plasmonic nanoparticles. When metal surfaces are irradiated with light, which is known to be an electromagnetic wave, there is an oscillation of the free electrons because of the formation of a dipole. These combined oscillations are defined as plasmons. In the dipole so formed, the electrons migrate to restore its initial configuration, but the electromagnetic waves which still oscillate force the electrons of the material to oscillate at the same frequency as that of the irradiating light (Figure 1). This is when the fundamental condition of resonance is achieved. The prerequisite is that the wavelength of the irradiating light has to be greater than or equal to the frequency of the plasmons. The easiest and most convenient way to modulate the plasmon resonance of a metal nanoparticle is to engineer the shape and size of the particles or shelling it with a non-conducting material like silica of varying thicknesses. Randomly distributed arrays of homogenous or heterogeneous nanostructures with variable sizes ranging up to tens of nanometres are much smaller than the wavelength of the interacting electromagnetic radiation and, thus, provide further scope of modulation in their optical properties (Lakowicz, 2005; Lakowicz et al., 2008; Ray et al., 2009a; Szmacinski et al., 2009; Wurtz et al., 2011; Li et al., 2017; Nicholls et al., 2017; Vestler et al., 2018).

**FIGURE 2:**
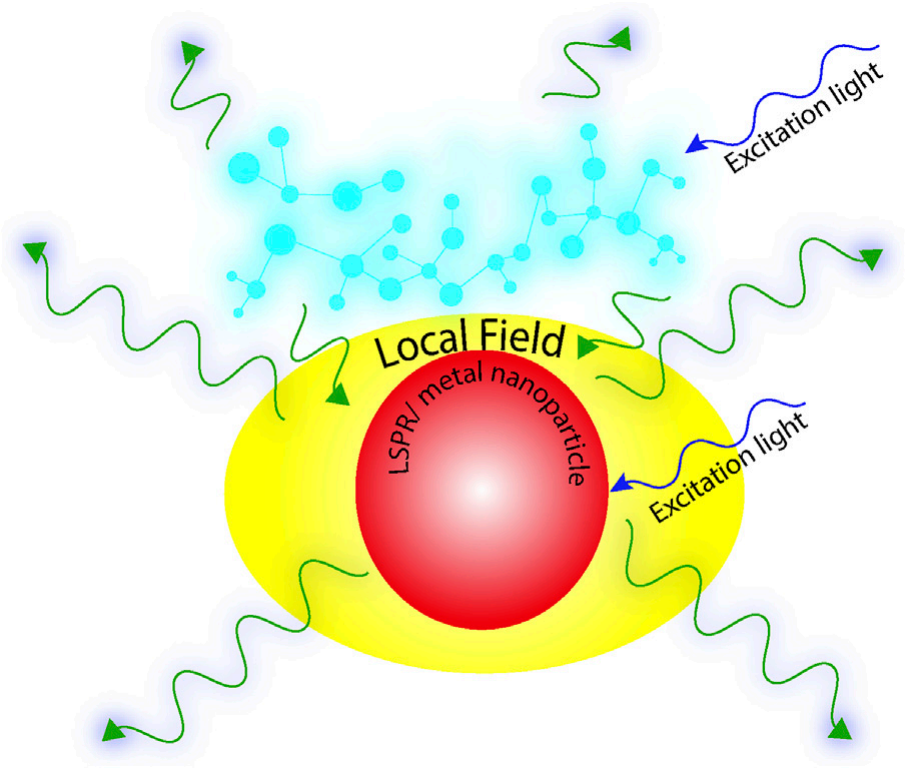
Schematic representation of plasmon-enhanced fluorescence (PEF).

Now, if a fluorophore molecule comes in the vicinity of the plasmonic nanoparticles, then there might arise a condition where resonance is achieved between the frequency of the fluorophore emission and that of the plasmon resonance. Under such circumstances, because of elastic scattering, an enhanced emission at the same frequency as that of the fluorophore could be observed. This phenomenon is defined as plasmon-enhanced fluorescence (Figure 2). Ideally, a significant overlap between the LSPR of the metal nanoparticle and the absorption/emission spectra of the fluorophore is a prerequisite condition to achieve optimal PEF (Lu et al., 2011). With an aim of providing amplified signals for advanced techniques like surface-enhanced Raman scattering (SERS), tip-enhanced fluorescence (TEF), and other single-molecule fluorescence methods, this tool has proved its omnipresence (Hartschuh, 2008; Schmid et al., 2013; Li et al., 2021; Sim et al., 2022; Li et al., 2023). As discussed earlier, the entire phenomenon of plasmon-enhanced fluorescence is based on the fundamental principle of the enhancement of the electromagnetic field, owing to the coupling of the incident light frequency with the frequency of the surface plasmons, and it has a very strong dependence on the shape, size, and interparticle separation distance in the metal nanostructures (Laible et al., 2021; Yang et al., 2023). There are specific domains within the nanostructures where the electric fields are intensely confined within the noble metal (Au, Ag, etc.) nanostructures, and these regions are defined as plasmonic “hotspots.” Engineering the interparticle gap proved to be an efficient pathway to modulate the plasmon resonance and, consequently, the electromagnetic fields. It has already been established that with the decrease in interparticle gap size, the localized electromagnetic field shows an exponential increase. Since this interparticle gap and topography play a vital role in LSPR, this gap has sparked significant interest in recent years (Laible et al., 2018; Dai et al., 2020; Laible et al., 2020).

The distance between the fluorophore and the metal nanoparticle plays a vital role in PEF (Ray et al., 2007). Before the advent of PEF, it was widely established that when metal surfaces are in close proximity of excited fluorophores, there is an ample chance that the excited fluorophores lose their excess energy by availing a non-radiative channel, i.e., resonance energy transfer, and as a consequence, quenching could be observed (Alivisatos et al., 1987; Yeltik et al., 2013; Wang et al., 2020a). In a recent report, this phenomenon of quenching in the presence of metal nanoparticles of Pd was successfully exploited to develop and design the ultrasensitive detection of fluorescently labelled DNA and proteins (Li et al., 2015). In some cases, a fluorophore attached directly to a metal surface may form an entirely separate entity with different photophysics altogether. The surface plasmon-enhanced electromagnetic field hence presents a situation where the PEF may be superseded by surface plasmon-induced quenching, and hence, an optimization is necessary. Thus, an appropriate spacer of specific thickness is needed to balance these two counterintuitive phenomena. In another recent report, polyelectrolyte multilayers were efficiently used as spacers to achieve enhanced fluorescence signals from lanthanide-doped conversion nanoparticles (UCNPs) with AuNRs. The enhancement in emission was demonstrated to be dependent on both the thickness of the dielectric polyelectrolyte multilayer spacers and the size of the AuNRs (Feng et al., 2015). Another contemporary report demonstrated that for randomly oriented fluorophores, maximum enhancement in fluorescence could be observed for substrate side detection with spacers having a low refractive index viz. Teflon, SiO_2_, etc. (Akimov and Sun, 2017). The discovery of surface plasmon polaritons in the 1950s provided the necessary impetus that led to the development of SERS in the mid-1970s (Fleischmann et al., 1974). The last couple of decades have witnessed a significant growth in this field, with applications demonstrated in various domains viz. optical and photovoltaic devices, bioanalytics, etc. (Liu et al., 2018; Spitzberg et al., 2019; Peng et al., 2022a; Kumar et al., 2022). This review primarily focusses on the bio-analytical applications of plasmonic nanoparticles in fluorescence in the last 20 years.

## 2 Basics of plasmon-enhanced fluorescence

When an ensemble of molecules is irradiated with light, a certain fraction of the molecules residing in the ground state absorbs the energy and is promoted to the higher excited states. Excited-state depopulation occurs mainly by two pathways, namely, radiative and non-radiative. The non-radiative pathways, which usually involve internal conversion (IC) and intersystem crossing (ISC), are usually faster and occur in the femtosecond–picosecond time regime. Emission efficiency or fluorescence quantum yield is defined as the ratio of the radiative rate constants and sum of all rate constants (Eq. 2). Physically, it is a measure of the probability of the depopulation of the excited state by fluorescence rather than any other non-radiative pathway (Eq. 1).

$$\phi_{f}^{sample}=\phi_{f}^{reference}.\frac{\frac{F_{sample}}{1-10^{-A_{sample}}}}{\frac{F_{reference}}{1-10^{{-A}_{reference}}}}.\;{\left(\;\frac{n_{sample}}{n_{reference}}\right)}^{2}\;\ldots ..\text{eqn},$$ (1)

$$\phi_{f}=\frac{k_{r}}{k_{r\;}+k_{nr}}\;\ldots \ldots \text{eqn},$$ (2)

where *F*_*sample*_ and *F*_*reference*_ are the integrated emission intensity and *A*_*sample*_ and *A*_*reference*_ are the absorbance at the excitation wavelength of the sample and reference, respectively. $\phi_{f}^{ref}$ is the emission quantum yield of the reference. *n* is the refractive index of the medium used for sample and fluorescence. *k*_*r*_ and *k*_*nr*_ are the radiative and non-radiative rate constants, respectively.

Fluorescence lifetime is another intrinsic parameter which is defined as the average time a molecule spends in the excited state. It is mathematically coined as the reciprocal of the sum of all rate constants (Eq. 3):

$$\tau_{f}=\frac{1}{k_{r\;}+k_{nr}}\;\ldots \ldots \text{eqn}.$$ (3)

For dilute solutions, the Lambert–Beer law holds good, i.e., absorbance is a linear function of concentration, i.e., A=ϵcl= log P_0_-log P, where A is the absorbance or optical density, ϵ is the molar absorption coefficient, *c* is the concentration of the sample, *l* is the path length through which the light traverses, and P_0_ is the excitation power. Emission intensity, which is defined as F=Q P_0_ (1–10^−*ϵcl*^), is directly proportional to the power of the excitation light and the concentration of the sample until an inner-filter effect crops in. The point is that quantum yield and lifetimes are better parameters than intensity since both are independent of the power of excitation light.

When the plasmon nanoparticle–fluorophore conjugate is irradiated with light of a specific wavelength having power P_0_, both the metal nanoparticle and the fluorophore absorb the energy, which results in a molecular excited state of the fluorophore and an LSPR in the plasmon nanoparticle, provided the conditions discussed above are met. Now, if the placement of the molecule is beyond the energy transfer distance, then possibilities of excited-state relaxation by non-radiative pathways like Forster resonance energy transfer (FRET) are eliminated. If the electromagnetic local field generated by the LSPR couples with that of the fluorophore, PEF could be observed. This enhancement in near-field intensity (E) leads to a change in the excitation power <|E|^2^. P_0_> in the region of the local field, which, consequently, results in an increase in the radiative rate constant (*k*_*r*_) (Aroca, 2006; Koya et al., 2021). Thus, experimentally, the parameter we end up recording is enhanced emission intensity concomitant with a decrease in fluorescence lifetime (τ_f_) in the near-field region of the plasmonic nanoparticle. On the contrary, in the far field, scattered frequencies from the plasmons interact with frequencies of the emitters, resulting in an enhanced electromagnetic field.

## 3 Fabrication of plasmonic nanomaterials

The role of shape, size, and interparticle distances in influencing the plasmonic hotspot and, in turn, the enhanced electromagnetic field has been briefly discussed in the *Introduction* section of this report. In order to achieve tunability and often periodicity in the placement of the metal, atoms play a vital role. Attaining nanometer‐scale sensitivity demands state-of-the-art fabrication technologies.

### 3.1 Top-down approaches

#### 3.1.1 Electron beam lithography

**FIGURE 3:**
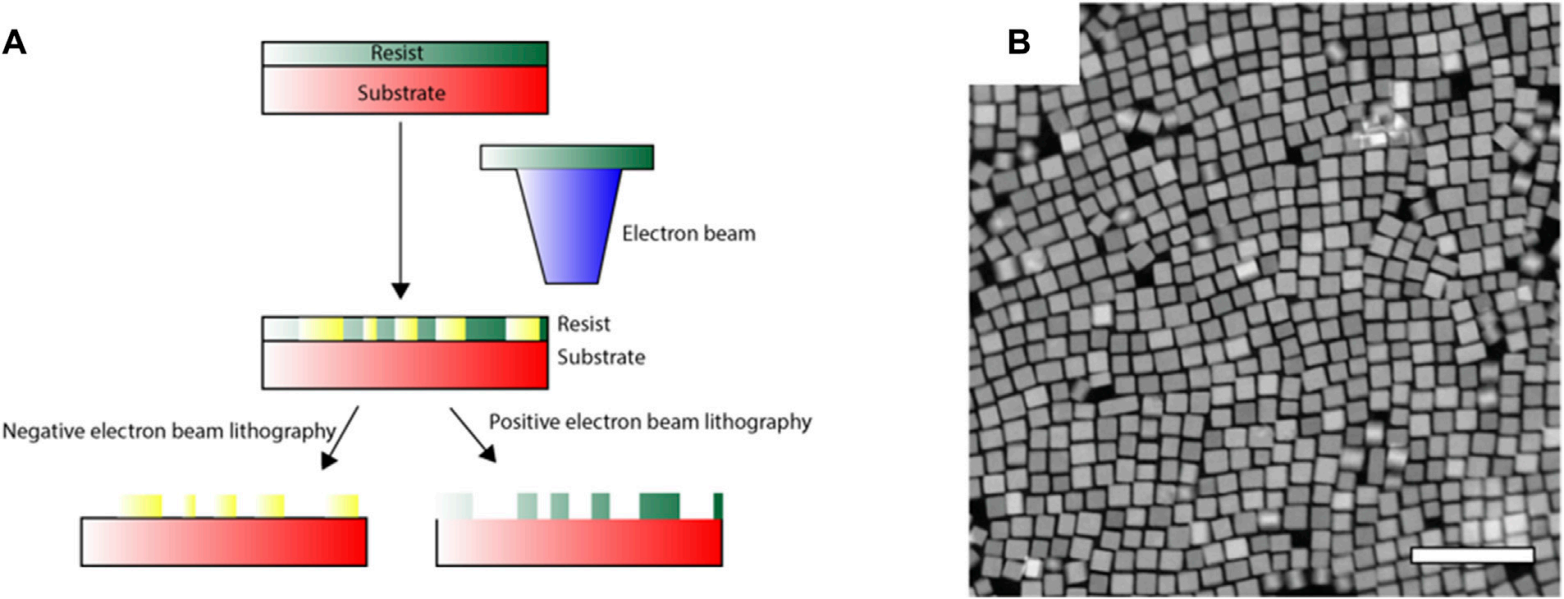
**(A)** Schematic representation of the electron beam lithography (EBL) technique. **(B)** Example depicting structural modifications in FSn:In_2_O_3_ nanoparticles using EBL (Roccapriore et al., 2022).

Conventional lithographic techniques have limitations in spatial resolution, which restricts their use in fabricating subwavelength-scale features, a necessity for their use in the visible-wavelength domain. The diffraction limit of light used in conventional photolithography is usually in the order of hundreds of nanometres, which in electron beam lithography (EBL), significantly improves to tens of nanometres, allowing the fabrication of detailed structures (Qin et al., 2021; Roccapriore et al., 2022). After covering the surface of the substrate with a resist, the electron beams are allowed to impinge on specific areas of the sample that results in directing writing on the resist layer (Figure 3A). Part of the resist layer is then etched with a developer viz. acetone. A subnanometer metal film is then deposited on the etched structure, followed by lifting-off to obtain the metal plasmonic nanomaterial (Figure 3B).

#### 3.1.2 Focused ion beam lithography

In focused ion beam (FIB) lithography, heavy-mass atoms viz. Ga are usually used to bombard the substrate surface for direct-write patterning. FIB mainly relies on high-energy, short-range secondary electrons instead of both the primary and secondary electrons, which plays a role in EBL. Thus, “resists,” which constitute a very important component of EBL, are not required in this case. The ions which are used in this method are massive compared to electrons used in EBL. Hence, the wavelength is smaller, which consequently results in less scattering than in the former. It can be used to directly mill a pattern on a substrate for prototyping of plasmonic nanomaterials (Morimoto et al., 1986). However, in some cases, there is Ga ion contamination that alters the very nature of the plasmonic nanomaterials. Recent technologies using He- and Ne-based FIBs have been proven to be successful in reducing this issue (Boden et al., 2011).

#### 3.1.3 Direct laser writing

Direct laser writing (DLW) or multiphoton lithography relies on the principle of two photon absorptions that induce a drastic change in the solubility of the resist. In this case, well-desired structuring is carried out by irradiating photoresists with a monochromatic beam of light. It serves as a diverse, efficient technology for the fabrication of sub-micron-resolved 3D nanostructures (Bernardeschi et al., 2021). Coupling DLW with electrochemical metal deposition serves as a very efficient methodology to fabricate complex 3D plasmonic nanomaterials. However, a point to note in this case is the spatial resolution that can be achieved here, i.e., 100–150 nm. However, when combined with stimulated emission depletion microscopy, the best achievable resolution can be up to 50 nm (Fischer and Wegener, 2011; Elmeranta et al., 2016).

### 3.2 Bottom-up self-assembly approaches

In addition to top-down approaches, another relatively convenient way of the preparation of plasmonic materials involves the use of building blocks in order to achieve complex nanostructures of homogenous chemical composition in the solution itself. Molecular interactions like hydrogen bonds, van der Waals forces, and stacking interactions constitute the major forces that cause self-assembly of the building blocks at the nanoscale. Because of their much lower cost and intrinsically additive nature, bottom-up approaches have been widely used in the past decade for the fabrication of plasmonic nanomaterials (Ozin et al., 2009; Tang et al., 2020). Plasmon coupling is a process which heavily relies on the spatial arrangement of the nanostructures within the assembly, and hence, a very brief discussion on the complex interplay of underlying entropic and other interparticle forces mentioned becomes relevant in this context. Some of the commonly used bottom-up approaches involve sol–gel processing, chemical vapour deposition (CVD), laser pyrolysis, and colloidal self-assembly. The sol–gel process involves the mixing of dispersed solid nanoparticles in a homogenous liquid to invoke the formation of three dimensional agglomerates of specific morphologies (Kumar et al., 2019). Electrotuneable voltage-controlled self-assembly of plasmonic nanoparticles at the interface of two immiscible electrolyte solutions was successfully evoked to obtain nanoplasmonic liquid mirrors (Montelongo et al., 2017). In CVD, one of the substrate surfaces is exposed to a volatile precursor to obtain nanomaterials of varied shapes, sizes, and thicknesses. Using vanadyl acetylacetonate (VO(acac)_2_) powder as a volatile precursor, nanomaterials with different morphologies were prepared utilizing CVD (Wang et al., 2010). Trimeric and heptameric clusters of Au nanoshells exhibiting pronounced Fano resonance were obtained on drying polymer-coated nanoshells (Fan et al., 2010). In colloidal self-assembly, for example, the plasmonic nanoparticles are usually capped by a layer of passivating ligands known as stabilizers or capping agents, which prevents chemical changes. There is a solvation layer surrounding the nanoparticles because of electrostatic interactions, and these interactions counter the van der Waals attractive forces (Bishop et al., 2009; He et al., 2012; Li et al., 2020). The mechanism of the aggregation of plasmonic nanoparticles is analogous to polymerization reactions. In some cases, oligomers that are formed from the aggregation of individual plasmonic monomers serve as the repeating unit, whereas in others, particle assembly at very low rates favours long-chain formation, following the chain growth pathway. It has been found that the side bonding sites on the individual repeating units of the plasmonic oligomers are more than the terminating sites, and hence, side chain polymerization becomes kinetically favoured (Wang et al., 2012). These techniques for the preparation of plasmonic nanoparticles directly from solution have been extensively used over decades. In a recent report, using an electrochemically driven self-assembly process, trimeric Au nanolenses having an interparticle size of sub 2 nm were synthesized (Lloyd et al., 2017). The first report on the synthesis of Al nanocrystals using oleic acid as the capping agent and (CH_3_)_2_C_2_H_5_NAlH_3_ as the precursor varied the relative proportion of the solvents viz. tetrahydrofuran (THF) and dioxane to achieve size tunability (McClain et al., 2015). The concept again is to moderate the electrostatic interactions operating out of solvation and the van der Waals forces (Jacobson et al., 2020). In recent days, modified DNA structures are widely being used as templates for the synthesis of plasmonic nanomaterials (Liu and Liedl, 2018; Wang et al., 2020b). In a seminal work by Rothemund, a 7-kilobase single-stranded DNA scaffold was efficiently twisted into several 2D nanostructures using a technology which he termed as “DNA origami” (Rothemund, 2006).

## 4 Plasmonic nanomaterials: role of metals

Plasmonic nanomaterials have generated significant interest in the past decade because of their diversified applications, owing to plasmonic coupling spanning the entire wavelength, i.e., from ultraviolet to infrared (Alivisatos et al., 1987). It has been discussed earlier (Section 2) that in plasmonic nanostructures, free-space electromagnetic energy is confined to ultrasmall regions defined as “hotspots.” The LSPR can be effectively tuned by engineering the size and structure of the nanomaterials (viz. nanoparticles of various sizes and shapes, nanodiscs, nanorods, etc.) and also the dielectric properties of the metals. Because of the ease in the modulation of the LSPR, the optical properties can also be tuned to cover the entire range of the spectrum. Nanoporous gold nanomaterials had been, for a very long time, used for tunability in the visible-to-IR range. Varying the size of a monolayer of polystyrene beads over Au nanodiscs prepared by the deposition of gold and silver alloys on a Si wafer helps in tuning the diameter of the Au nanoparticles (Zhao et al., 2014). In another report, a silver halide electroreduction process was utilized to achieve tunability in pore size and diameter (Seok et al., 2018). However, in recent days, the focus is on Al-based plasmonic nanostructures owing to their natural abundance and long range of tunability, ranging from the UV to the IR region of the spectrum (Jacobson et al., 2020). However, preparation of size-controlled Al nanocrystals did not receive initial impetus, owing to the very high reactivity of this alkaline earth material in the presence of ambient oxygen (O_2_) and water (H_2_O). Hallas et al. synthesized crystalline Al nanocubes by using a transition metal catalyst viz. Tebbe’s reagent with an excess of AlH_3_ in THF. This was one of the first reports that expanded the use of a conventional transition metal catalyst in tuning the shape of metal nanoparticles (Clark et al., 2019). Al may prove to be a successful alternative to Au and Ag plasmonic nanomaterials, owing to its natural abundance and higher plasmon tunability (Tian et al., 2017). However, the unstable nature and very high reactivity have impeded the real-time applications of these plasmonic nanomaterials. Another metal that has been investigated very recently in this line is rhodium (Rh). Clavero et al. demonstrated chemical reduction on self-assembled micelles with trisodium hexachlorodate (Na_3_RhCl_6_) as the Rh precursor to obtain nanoporous Rh nanostructures (Clavero, 2014).

## 5 Substrates for PEF

The first report of PEF involved the enhancement of the emission intensity of fluorescein isothiocyanate (FITC) and rhodamine 6G on rugged silver islands (Chen et al., 1980). Later studies by Glass et al. delineated the role of the degree of overlap of the emission and absorption spectra on the enhancement of the fluorescence intensity (Glass et al., 1980). Following these developments, many different fabrication techniques were brought to light. We broadly categorize these into two subsections. The first section provides a brief overview of the physical modifications that have been effective in bringing about PEF. The second section elaborates a few of the wet chemical techniques that are broadly being used.

### 5.1 Physical modifications

#### 5.1.1 Metal islands and hybrid nanostructures

**FIGURE 4:**
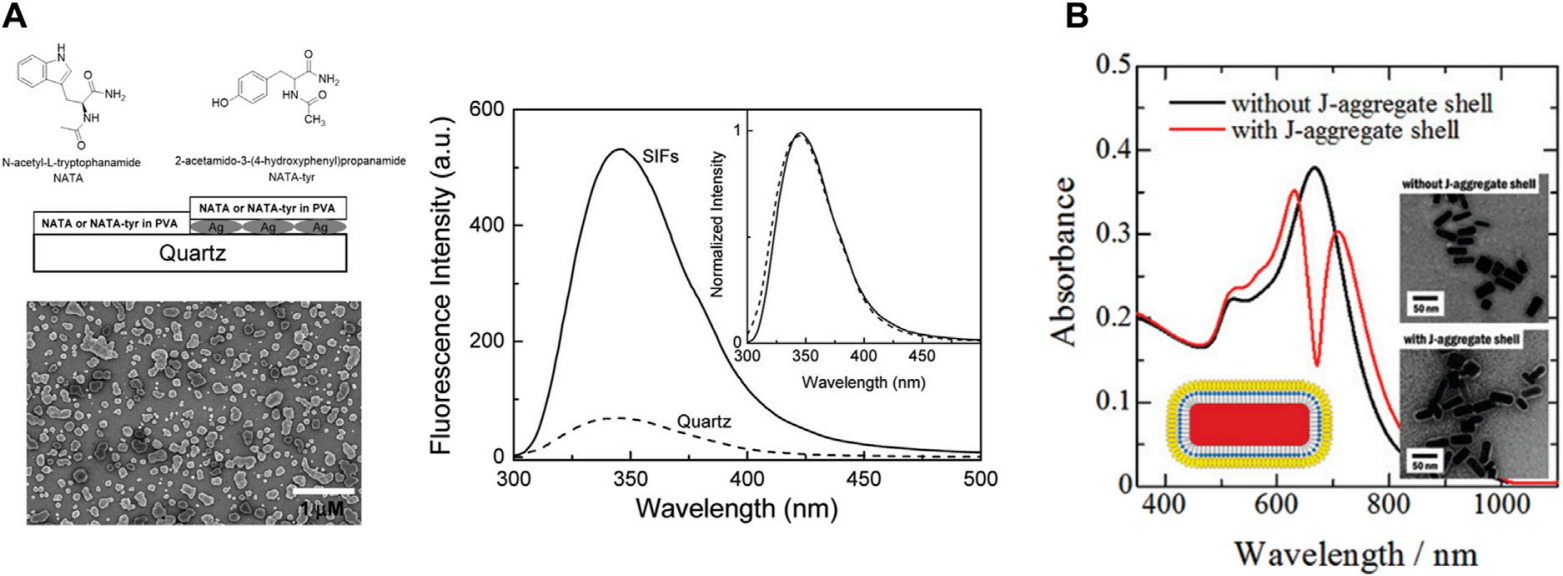
**(A)** Emission spectra of the 15-nm PVA film containing NATA on quartz and silver island films (SIFs) (Ray et al., 2008a). **(B)** Change in the absorption spectra of shelling Au nanorods with J-aggregated cyanine dyes (Yoshida et al., 2009).

PEF for fluorophores emitting in the visible region of the spectrum has been widely studied using silver nanoparticles. In one of the initial reports on the enhancement of tryptophan emission in proteins, the enhancement of the emission intensity of *N*-acetyl-1-tryptophanamide (NATA), a tryptophan analogue could be observed close to the Ag nanostructured surfaces (Figure 4A) (Ray et al., 2008a). In one of the initial reports on the enhancement of the emission of semiconductor quantum dots in the presence of plasmonic nanoparticles, Ray et al. demonstrated a several-fold enhancement of the emission intensity of CdTe quantum dots on silver island films (SIFs). This, coupled with single-molecule blinking experiments, delineated the intricacies of heterogeneity in the emission enhancement on glass and SIF surfaces (Ray et al., 2006).

#### 5.1.2 Plasmonic nanoparticles with a capping layer

The spatial proximity between the fluorophore and the plasmonic nanoparticles is a vital factor to induce PEF (Section 2). The easiest way to achieve that is to deposit a layer of the fluorophores on the plasmonic surface. However, there are a couple of drawbacks in this case; first, there is always a possibility of Forster resonance energy transfer leading to quenching instead of enhancement (Section 2) (Glass et al., 1980). Second, a simple mixing might lead to the formation of an entirely different species. To prevent these, plasmonic nanoparticles are coated with a layer of organic polymers, which prevents them from coalescing. It has a major influence on the self-assembly process of the plasmonic nanomaterials and also helps in modulating the distance between the plasmonic surface and the exciton material. In a recent report, the role of capping layers was demonstrated using a library of gold nanourchins (NUs), where the observed coupling values were higher in the presence of the capping layer. The formation of the plexitonic hybrids was also significantly influenced by the extent of dye aggregation (Peruffo et al., 2021). Yoshida et al. efficiently synthesized hetero-nanostructures consisting of a Au-nanorod core with an inner spacer and an outer spacer of J-aggregated cyanine dye. They demonstrated that such double-layered composite structures provide flexibility in controlling the plasmon–exciton coupling (Figure 4B) (Yoshida et al., 2009).

### 5.2 Wet chemical modifications

#### 5.2.1 Nanoprisms

**FIGURE 5:**
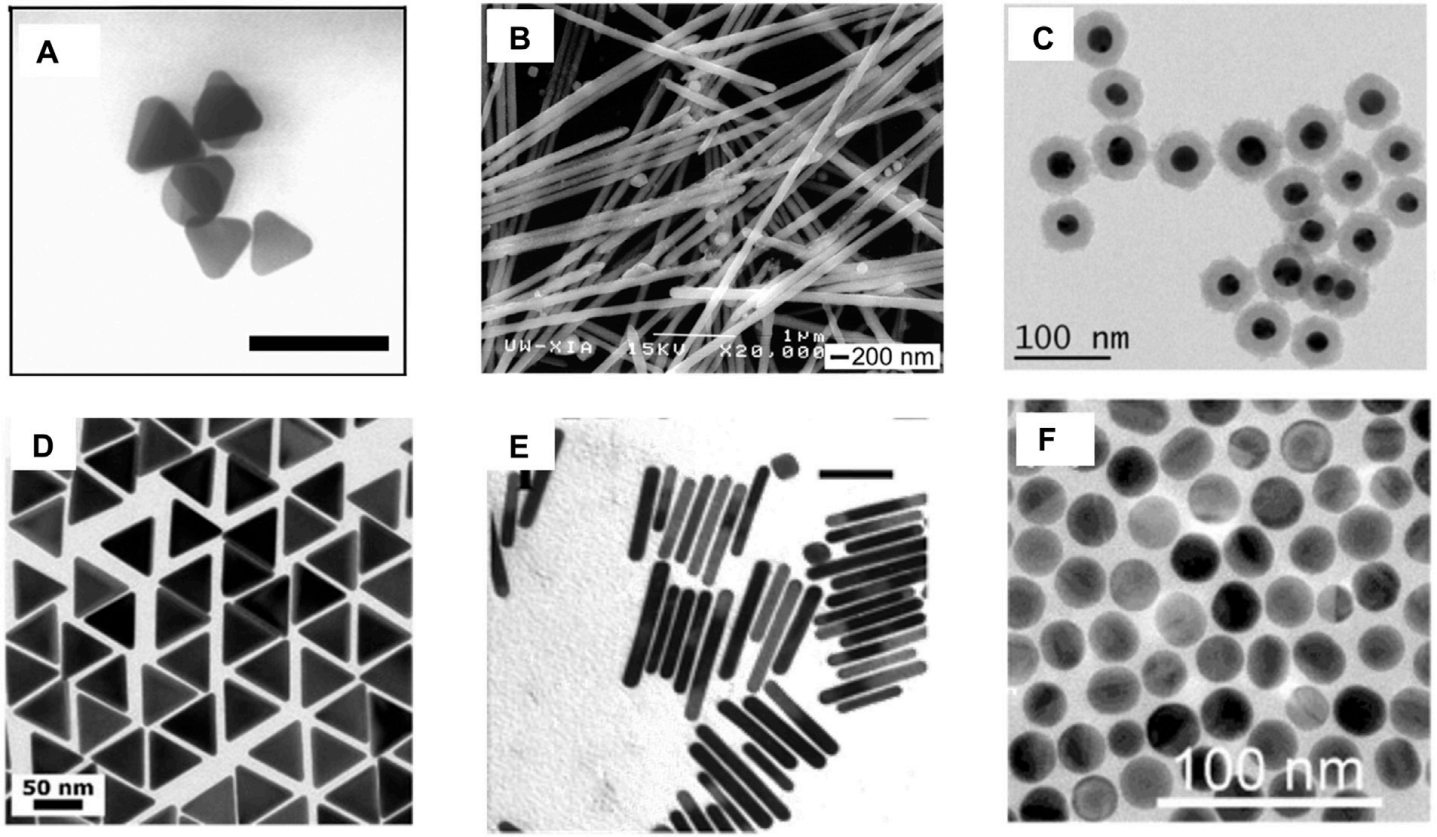
**(A)** Ag nanoprisms having an average lateral dimension of 40 ± 5 nm and an average thickness of 8.5 ± 1.4 nm; the bar corresponds to 100 nm (Frank et al., 2010). **(B)** SEM images of Ag nanowires with *n*_PVP_/*n*_AgNO3_ = 1.5 (Sun et al., 2002). **(C)** TEM images of AuNP@SiO_2_ of uniform thickness (Gogoi et al., 2021). **(D)** TEM images of 60-nm gold nanotriangles (Scarabelli and Liz-Marzán, 2021). **(E)** TEM images of gold NRs with plasmon band energies at 1,250 nm; the bar corresponds to 50 nm (Nikoobakht and El-Sayed, 2003). **(F)** TEM AuNS with diameters 24 ± 2 nm (Ruan et al., 2014).

In a simple, robust, and cost-effective technique, silver (Ag) nanoprisms were reportedly synthesized by reducing Ag^+^ in AgNO_3_ using a solution of sodium borohydride (NaBH_4_) (Figure 5A). The reaction time and colour were indicative of the size of the nanoprisms (Frank et al., 2010). Other photochemical techniques can also provide a meticulous control of the prism size.

#### 5.2.2 Nanowires

The Ag nanowire is another exotic plasmonic nanocrystal that has been used widely in various applications. One of the initial reports of synthesis demonstrated a seed-assisted route, where a platinum (Pt) nanoparticle seed was first synthesized by the reduction of platinum chloride (PtCl_2_) with ethylene glycol, followed by the addition of AgNO_3_ and polyvinyl pyrollidone (PVP), which allowed the formation of Ag nanoparticles. The pre-synthesized Pt nanoparticles acted as the template for the epitaxial growth of the Ag nanoparticles. When these nanoparticles were heated at high temperatures, because of Ostwald ripening, they could not retain their spherical shape and gradually started forming wires of size ranging from 30 to 40 nm (Figure 5B). A meticulous control over the size of the wires could be achieved by varying the reaction temperature and also the proportion of PVP to AgNO_3_ (Sun et al., 2002).

#### 5.2.3 Core–shell plasmonic nanomaterials

In core–shell nanomaterials, the core (typically a metal nanoparticle) is coated by another dielectric compound, which prevents the core from coming into direct contact with the surrounding environment and, hence, increases its stability. This broadens its scope by enabling the modulation of the plasmonic properties of the core. Optically transparent SiO_2_ is one of the widely used shelling materials. The prerequisites of an ideal shelling material include a uniform, ultrathin layer that prevents the plasmonic core from coming into direct contact with the solution. Using (3-aminopropyl)trimethoxysilane (APTMS) as the linker and Na_2_SiO_3_ as the Si precursor, thin (2 nm) shelling was demonstrated efficiently at higher temperatures (100 C). The reaction duration was varied to obtain shells of varied thicknesses (Krajczewski and Kudelski, 2019).

#### 5.2.4 Nanotriangles

In recent years, truncated triangular pyramid-shaped plasmonic nanomaterials have attracted significant interest, owing to the anisotropic distribution of the LSPR and generation of a strong electromagnetic local field, which consequently results in spectral tunability from the visible to the IR domain (Section 2). In a recent report, gold nanotriangles (AuNTs) were synthesized using the time-tested seeded growth technique in which Au (III) in HAuCl_4_ was first reduced to Au using NaBH_4_ as the reducing agent. Subsequently, the Au nanocrystal seeds thus obtained were aged and diluted in a cetyltrimethylammonium chloride (CTAC). The seeds were subsequently transferred into two growth solutions of CTAC, NaI, and HAuCl_4_, which ensures the formation of AuNTs (Figure 5D) (Scarabelli and Liz-Marzán, 2021).

#### 5.2.5 Nanospheres and nanorods

The most widely used plasmonic nanomaterials in biomedicine and photonic devices involve the use of spherical gold nanoparticles (AuNPs) and nanorods (AuNRs) (Ruan et al., 2014; Zheng et al., 2021). The reason behind the choice is their ease of synthesis, stability, and fairly strong LSPR couplings in the visible and NIR ranges. However, AuNRs have spherical symmetry, which limits their LSPR response only to the visible range. This problem was, however, bypassed in AuNRs, where, because of anisotropic LSPR, increased flexibility in the modulation of the LSPR could be achieved. In a modified seeded growth method, AuNPs were prepared with a surfactant CTAB in the seed formation phase, followed by a growth solution in which the Ag content was changed to obtain NRs with modulated aspect ratios (Nikoobakht and El-Sayed, 2003).

## 6 Examples of fluorescence enhancement

### 6.1 Role of hyperbolic metasurfaces in the enhancement of spontaneous emission

**FIGURE 6:**
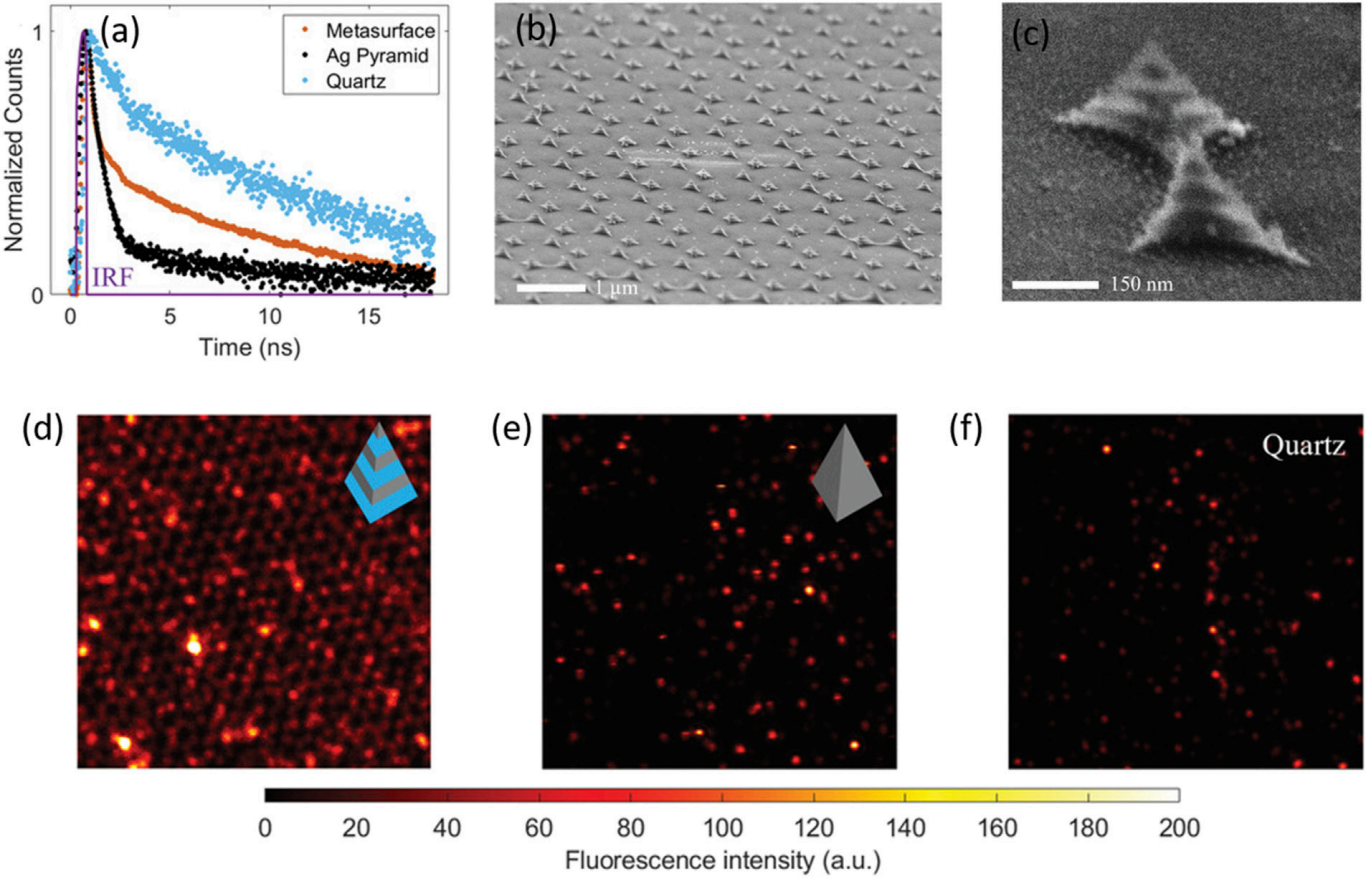
**(A)** Temporal parameters of the pyramidal metasurface: **(A)** fluorescence decays of the nitrogen vacancies (NVs) on different surfaces: black (Ag pyramid), blue (quartz), and yellow (pyramidal heterostructure) **(B,C)**. SEM images of the fabricated heterostructures at different magnifications **(D,E,F)**. The fluorescence intensity images of NV centres measured on the pyramidal metasurface, the Ag nanopyramid, and the quartz substrate, respectively, in a dimension of ≈14 μm × 14 µm with an excitation wavelength of 532 nm (Zheng et al., 2023).

Nitrogen vacancy (NV) sites in nanodiamonds are a distinct example of solid-state nanoemitters displaying a broadband emission spectrum. Owing to their significant stability, they can serve as a unique and robust single photon source. The spin-selective optical transitions in nanodiamonds diversify their scope of applications in various fields. However, the extraction of the generated photon from the NV centres becomes a strenuous task, owing to longer fluorescence lifetimes and fewer numbers of photons. A recent report demonstrated the use of alternately layered, stacked silica–silver thin films in a pyramidal shape that aided in Purcell enhancement due to plasmonic coupling (Figure 6) (Zheng et al., 2023). In a contemporary report, resonance indicators of nanodiamonds and gold (RING) nanoassemblies were fabricated by modifying biotin-capped nanodiamonds and Au nanoparticles by the hybridization of complementary DNA sequences (Liang et al., 2023). It was demonstrated that the transition dynamics of the nitrogen-vacancy centres aids in higher local density of states and correspondingly enhances the oscillator strength because of a closed nanocavity. Using the concept of DNA assembly, RING nanoassemblies with different proportions of nanodiamonds and AuNPs were prepared to decipher the role of closed nanocavity in the enhancement of fluorescence of the NVs (Liang et al., 2023).

### 6.2 Applications in fluorescence imaging

**FIGURE 7:**
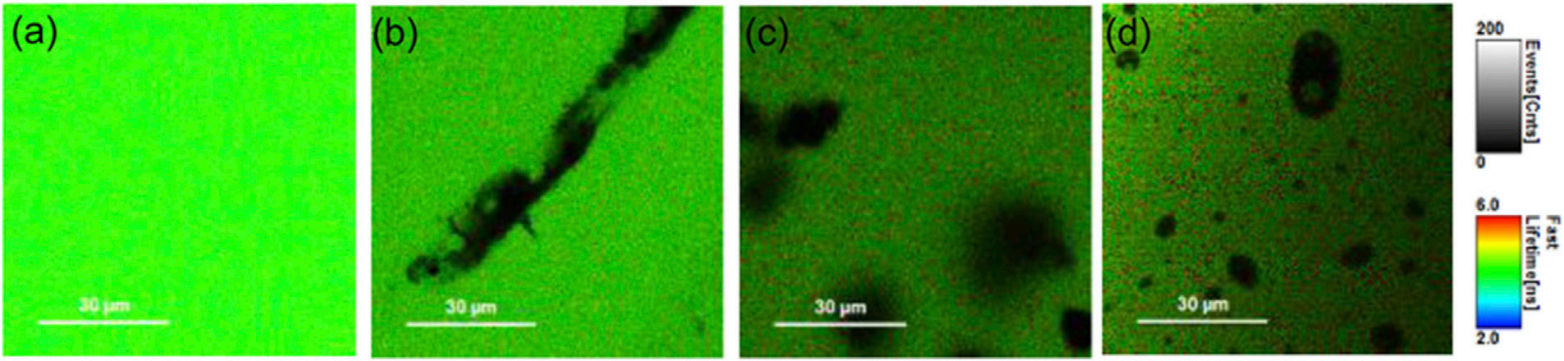
FLIM images of phantoms containing 50 μM fluorescein with **(A)** 0, **(B)** 2, and **(C)** 4 mg/mL of GNRs **(D)**. FLIM image of a phantom containing 50 μM fluorescein with 20 μg/mL of GNSs. The grayscale bar represents fluorescence intensity in counts/ms. The coloured scale bar displays the fluorescence lifetimes in nanoseconds (Fixler et al., 2014).

Fluorescence imaging is an excellent non-invasive method for the real-time monitoring of various physiological processes with the best spatial and temporal resolutions (Ntziachristos, 2010). However, small penetration depths and a moderate signal-to-noise ratio are an impediment to many such applications. The augmentation of the fluorescence signal because of the coupling of free electrons on the nanomaterial surface and the excited-state fluorophore not only leads to a plasmon-enhanced fluorescence but also contributes to better detection limits (Hong et al., 2014). Spatiotemporal properties of fluorescein-conjugated Au nanoparticles were delineated using a combination of fluorescence-lifetime imaging microscopy (FLIM) and direct reflectance (DR), which paved the way for multimodal bioimaging. A decrease in the fluorescence lifetime of fluorescein due to a change in the radiative rate constant in the near-field region of the AuNPs was successfully exploited to detect tumour surfaces (Section 2; Figure 7) (Fixler et al., 2014).

However, until very recently, the concept of plasmon-enhanced fluorescence for fluorescence imaging in the NIR region was limited. By the electrostatic assembly of fluorescent polymer dots on the surface of silica-coated gold nanorods, PEF could be observed. Since it was already established that PEF is a distance-dependent phenomenon, where the spatial distance between the plasmonic nanoparticle and the fluorescent probe plays a vital role, the thickness of the Si coating was varied to optimize the maximum enhancement (Anger et al., 2006). It was finally employed to probe the cerebral vasculature in live mice (Peng et al., 2022b). However, only two NIR probes, indocyanine green (λ_em_ = 828 nm) and methylene blue (λ_em_ = 686 nm) nm, have been clinically approved. So, the recent focus is on imaging in the short-wave infrared (SWIR) (900–1,700 nm) window. First, reduced autofluorescence and, second, lesser photon scattering have enabled SWIR imaging at significantly better depths within the tissue. Utilizing the concept of plasmon-enhanced fluorescence, commercially available SWIR dye IR-E1050-AuNR composites were successfully used for the *in vivo* imaging of ovarian cancer (Huang et al., 2021).

### 6.3 In molecular sensing

**FIGURE 8:**
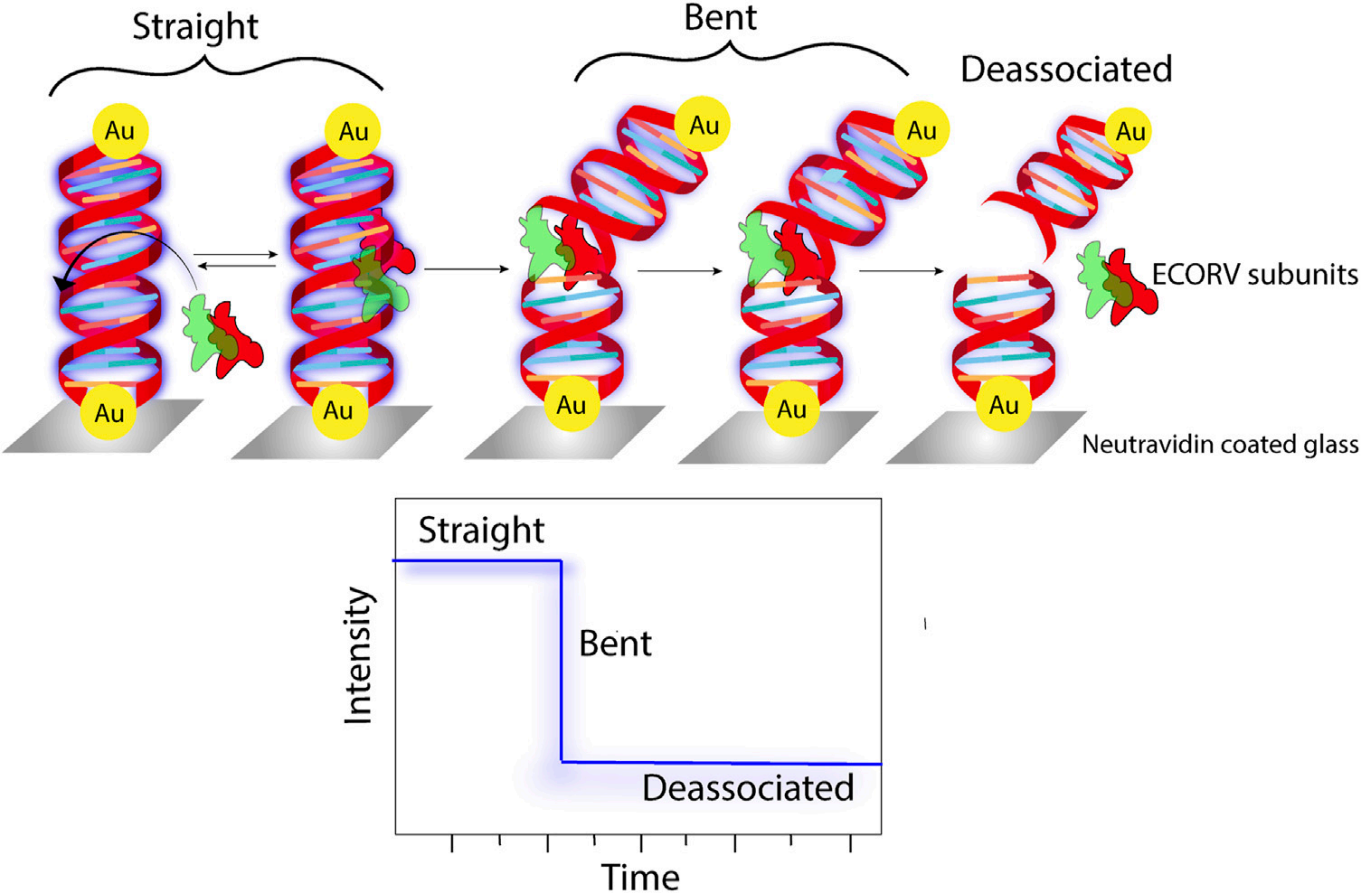
Schematic representation of fluorescence being used as a parameter to track the enzymatic cleavage of Au-tethered dsDNA (Reinhard et al., 2007).

PEF is a phenomenon that is associated with augmented fluorescence concomitant with an increase in the radiative rate constant. Using this very concept, PEF has been very effectively used in the past decade as a tool for sensing different analytes, biomolecules, nucleic acids, etc. (Gao et al., 2023; Lu et al., 2023; Rippa et al., 2024). The present technique of specific RNA quantification involves complementary base pairing between a target RNA and the nucleic acid probe. However, sensitivity and rapidity are two major challenges in this methodology. In a PEF-based technique, a target RNA is first annealed, followed by labelling with a fluorogenic probe, and finally adhered to the SIFs, where enhanced fluorescence was observed because of PEF (Aslan et al., 2006). Using fluorescence lifetime correlation spectroscopy (FLCS) as an efficient tool, a 5-fold enhancement in the emission of cyanine 5 (Cy5) was reported in the presence of 50-nm Ag colloidal particles (Ray et al., 2008b). In another report, the deposition of a 10-nm Ag film on Klarite, a commercially available SERS substrate, resulted in the 50-fold enhancement in fluorescence of streptavidin-conjugated Alexa 674 (A647) (Ray and Lakowicz, 2013). The biotin–streptavidin host–guest system immobilized on an Au surface showed an enhancement in the fluorescence of the labelled streptavidin moiety, owing to plasmon-enhanced fluorescence. Utilizing this concept, a unique DNA chip was engineered using a biotinylated catcher probe that can track the DNA-binding kinetics (Tawa and Knoll, 2004). Utilizing the same concept, a solid surface-based immunoassay was developed, which could detect human chorionic gonadotropin (hCG) hormone in serum with LOD as low as 0.3 mIU mL^–1^ (∼6 × 10^−13^ mol L^–1^) (Vareiro et al., 2005). An ultrasensitive biochip for surface plasmon-enhanced fluorescence assays was developed, which could detect IgG molecules at concentrations as small as 11 pM (Toma et al., 2013). Silver island films were used to enhance the fluorescence intensity of myoglobin immunoassay if labelled with the Alexa Fluor 647 dye (Matveeva et al., 2007). The concept of plasmon-enhanced fluorescence was exploited to develop an Au@polymer dot-based fluorescent immunoassay platform that can detect a prostate-specific antigen (PSA) with as less as 10 μL of the blood sample within 10 min (You et al., 2019). The proposition of thin Al films can also serve as an efficient substrate for metal-enhanced fluorescence with probes like NATA and tyrosine (Chowdhury et al., 2009). Another label-free approach of detecting tryptophan-containing proteins involved the augmentation of the tryptophan fluorescence in the presence of Al nanostructures (Ray et al., 2009b). In the last few years, there has been immense progress in the direction of label-free bioassays and sensing using the concept of plasmon-enhanced fluorescence (Figure 8) (Reinhard et al., 2007; Dai et al., 2022; Roy et al., 2023). The absolute quantification of membrane protein expression on cell surfaces is imperative to early cancer detection. For example, peptide–AuNP nanoprobes were designed and developed for the quantitative estimation of integrin GPIIB/IIIa, which, using NIR two-photon microscopy, could be directly visualized (Gao et al., 2015). Similarly, Ag–aptamer clusters were developed, which could provide significant quantitative insights into mIgM in live cells (Liu et al., 2014). In a recent report, a TiO_2_ cluster-based biosensing platform was demonstrated, which could track the expression levels of N-cadherin, an important biomarker of epithelial-to-mesenchymal transition (EMT) in malignant cells (Han et al., 2021). The use Au-Se-peptide nanoprobes in mapping the role of drugs viz. curcumin and 7-ethyl-10-hydroxycamptothecin in apoptosis in malignant cells was another significant advancement in this domain (Pan et al., 2019). The design and development of label-free assays is another field that attracts significant attraction. A highly controlled assembly of peptide-functionalized Au nanoparticles was demonstrated to be a sensitive label-free assay for the detection of blood coagulation factor XIII (Chandrawati and Stevens, 2014). The fabrication of a plasmonic nanogap cavity using colloidal Ag nanocubes on top of Au films resulted in field enhancements, which, when coupled with a PED4 assay, resulted in an ∼100-fold enhancement in fluorescence, which aided in the detection of an important cardiac biomarker viz. B-type natriuretic peptide (BNP) (Cruz et al., 2020).

### 6.4 Plasmon-enhanced fluorescence: DNA origami

**FIGURE 9:**
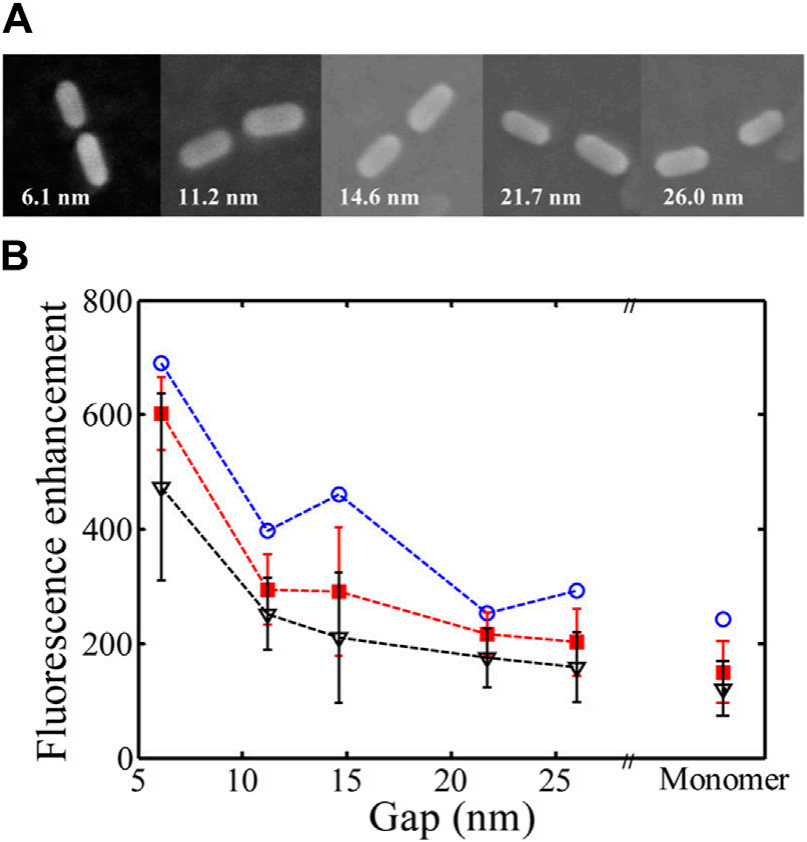
**(A)** SEM images of AuNR dimers with different gaps. **(B)** Dependence of fluorescence enhancement on distance (Zhang et al., 2015).

DNA origami is a technique based on the molecular self-assembly, which can serve as a template for the fabrication of discrete, complex nanostructures using a bottom-up approach. The complex interactions between the plasmonic nanoparticles and fluorophores can be engineered by immobilization on a 3D origami structure. The specificity of nucleic acid binding makes DNA origami an efficient tool for tuning the arrangement of plasmonic nanomaterials for enhanced emission. The DNA connector configuration plays a vital role in controlling the placement of a plasmonic particle. In the shear configuration, after antiparallel hybridization of the same terminal ends, a perpendicular orientation of the duplex with respect to the bound surface is achieved. On the contrary, in the zipper configuration, protrusion of the different terminal ends is observed, which, on binding, results in a tangential orientation. Schreiber et al. used hybrid nanostructures with varying distances between the fluorescent probe and metal nanoparticles to demonstrate a 1/*d* ^4^ distance-dependent quenching model (Schreiber et al., 2014). The use of Au nanoparticles for fluorescence enhancement is restricted to the NIR region, which was overcome by fabricating gold nanorod dimers on a DNA origami template. Using ATTO 655 as the fluorescent probe and engineering the distances between the nanorod tip and the probe, it was shown that an optimum distance of 6.1 nm was most favourable for achieving a 473-fold fluorescence enhancement (Figure 9) (Zhang et al., 2015). Similar studies were subsequently carried out using AgNPs having a higher scattering cross section (Vietz et al., 2017). In another report along the same line, different hybrid nanoparticle–DNA origami assemblies were used to explore the distance dependence on local field enhancement in monomeric and dimeric AuNPs (Pal et al., 2013).

### 6.5 Single-molecule detection

**FIGURE 10:**
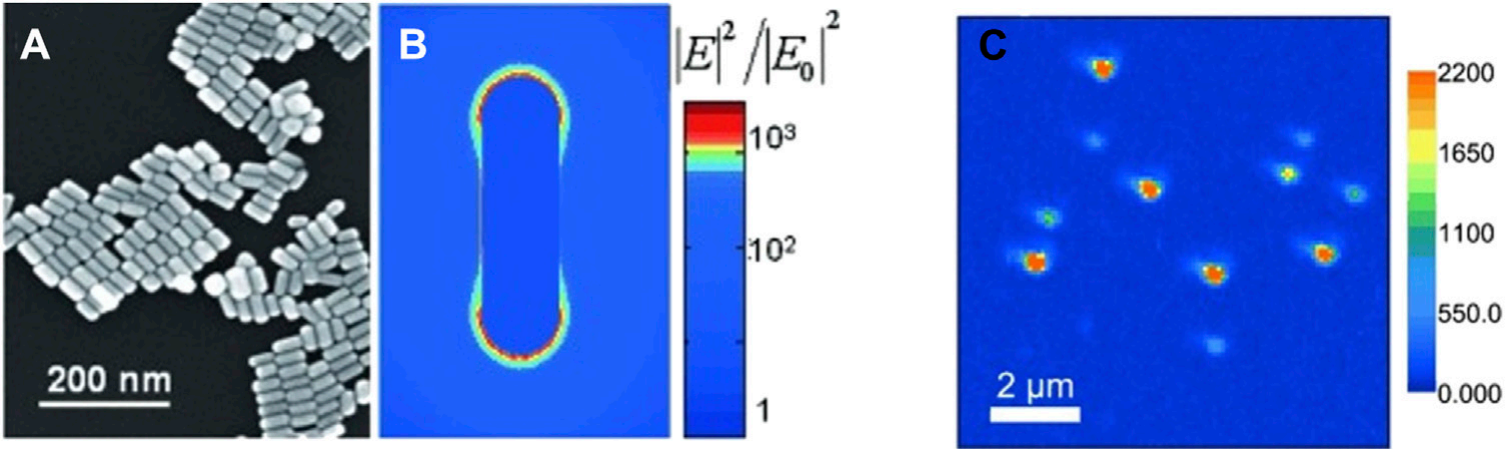
SEM image of **(A)** drop of the gold nanorod. **(B)** Map of the near-field optical intensity. **(C)** Single-molecule imaging of SMD enhanced by AuNRs (Yuan et al., 2013).

Another important domain where PEF finds significant use is single-molecule detection. One of the major issues with single-molecule fluorescence is the low signal-to-noise ratio. In biological systems, coupled with that is the issue of undesired analyte molecules contributing to spurious signals. Thus, the fluorescence from the target analyte must be isolated from the erroneous contributions (Ray et al., 2006; Ray et al., 2008c; Ray et al., 2015). In a seminal work using self-assembled dimeric nanoantennas (DNs) with a modified DNA origami template, higher fluorescence enhancement could be achieved compared to antennas fabricated by top-down lithography approaches. Even in the presence of a quencher like NiCl_2_, a 5,468-fold fluorescence enhancement with ATTO 647N could be observed, which aided in single-molecule detection at concentrations of 25 μM (Puchkova et al., 2015). In another work, Au bowtie nanoantennas fabricated using electron beam lithography on 50-nm-thick ITO-coated quartz coverslips were used for the fluorescence enhancement of a NIR emissive TPQDI probe. A 1,348-fold fluorescence enhancement was reported (Kinkhabwala et al., 2009). Orrit et al. reported the single-molecule fluorescence enhancement of crystal violet (CV) up to 1,100 times in the presence of AuNRs (Figure 10). The underlying concept is that a molecule prefers diffusing through the ideal position where the enhancement is maximum. Using high-fluorophore concentrations, the fluorescence enhancement of a single CV molecule in the presence of AuNRs was demonstrated (Yuan et al., 2013).

### 6.6 Devices based on plasmon-enhanced fluorescence

Small optical reading devices have recently proved to be indispensable, providing rapidity and cost effectiveness to certain diagnostic assays and conventional laboratory tests. Exploiting the computational capabilities and imaging abilities of commercially available smartphone devices, early diagnosis of diseases and quantitative detection of viruses like HIV and other chronic ailments have been developed recently (Inci et al., 2013; Rad et al., 2015; Nguyen et al., 2023). A handheld miniature microscopy device based on the principle of plasmon-enhanced fluorescence, coupled with a smartphone, could efficiently image 50-nm fluorescent beads. In the same report, the detection of 80 fluorophores was demonstrated in each diffraction-limited spot using the handheld microscope, which paved the way for the design and development of nanophotonic devices (Wei et al., 2017). An assembly of ZnO nanorod arrays in a microfluidic system, along with a photodetector, was successfully used for the point-of-care (POC) detection of cancer biomarkers viz. carcinoembryonic antigen, α-fetoprotein, etc. (Hu et al., 2013). A similar kind of assembly using ZnO nanorods with cyanine 3 (Cy3) and Cy5 served as an immunoassay for the detection of carcinoembryonic antigens (Liu et al., 2016). In brief, many different plasmonic nanostructures can be widely utilized in different biosensing platforms including point-of-care diagnostics (Semeniak et al., 2023). Plasmonic Au-nanoparticle platforms allow for the accurate detection and quantification of type 1 diabetes antibodies, overcoming the shortcomings of non-specific binding in ELISA, which makes it an important contender for point-of-care diagnostics (Zhang et al., 2014). A plasmonic gold nano-island chip was designed and fabricated for the efficient diagnosis of myocardial infarction (MI). The serum biomarkers viz. cardiac troponin I showed ∼130-fold enhanced NIR fluorescence in the presence of the plasmonic nanomaterial, which leads to quantitative detections with superior sensitivity to standard immunoassays (Xu et al., 2020).

## 7 Summary and perspective

This review delves into the recent advancements in plasmonic materials, with a specific focus on their ability to amplify fluorescence through near-field interactions and placing particular emphasis on the potential of utilizing plasmon-enhanced fluorescence for various photonic and analytical applications. The augmented spectral intensity in metal nanoparticles owing to the excitation of the LSPR results in the higher-extinction cross sections of the plasmonic nanoparticles, which leads to an enhancement in emission. This has been successfully employed in the domains of single-molecule detection, imaging, and the development of fluorescence-based biosensors. The theory will continue evolving and probe into the intricate details of molecular-level interactions between the plasmonic metamaterial and the quantum emitter. The past decade has witnessed a growth in the development of novel plasmonic materials, particularly focussing on semiconductors and conducting oxides. This exciting frontier holds immense promise for extending the spectral range of plasmonic effects beyond the limitations of traditional noble metals like gold and silver and other metals like aluminium. Accordingly, all of these plasmonic materials offer exciting possibilities for manipulating light across a broader spectrum of the UV-vis-IR regimes. This expanded range opens doors for innovative applications in the areas of enhanced biosensing and advanced optoelectronics. By enabling plasmonic interactions with biomolecules in UV-vis-IR regions, these materials could lead towards more sensitive and specific detection techniques for medical diagnostics. Tailoring the spectral response of plasmonic materials could potentially pave the way for the development of miniaturized and high-performance optical devices with functionalities like light modulation and switching.

Point-of-care testing is poised to benefit significantly from the development of planar plasmonic substrates. These plasmonic substrates can be fabricated using two main approaches: top-down nanofabrication techniques for precise patterning and the bottom-up nanoparticle assembly for potentially scalable production. Both approaches have shown promise in creating surfaces that enhance fluorescence for protein or DNA/RNA microarrays. This advancement could lead to rapid, portable diagnostic tools with increased sensitivity for detecting various diseases and biological markers. Despite a growth in research on plasmonic structures designed to enhance fluorescence for diagnostic and biosensing applications, there remains a significant gap between these promising tools and their translation into clinically relevant platforms that can effectively navigate the complexities of real-world biological systems. Even though plasmonic structures face some limitations, continued research and development hold promise towards notable improvements in the fields of photonics and optical sensing. By overcoming these challenges, a plethora of possibilities in manipulating light at the nanoscale can be accomplished, leading to advancements in the areas of ultrasensitive biosensing, ultracompact photonic circuits, and enhanced light-harvesting technologies.
